# Cardiopulmonary Resuscitation (CPR) Competency Retention Among Registered Nurses in Critical Care Versus General Care Unit

**DOI:** 10.3390/nursrep16080259

**Published:** 2026-07-27

**Authors:** Yahia AL-Helih, Majeda Al-Ruzzieh, Sami Al-Yatim, Mohammad Alawneh, Saleh Abual-Haija, Faten Odeh

**Affiliations:** 1 Faculty of Nursing, Middle East University, Amman 11831, Jordan; y.alhelih@meu.edu.jo; 2 Nursing Department, King Hussein Cancer Center, Amman 11181, Jordan

**Keywords:** CPR competency, competency retention, critical care, general care, nursing education

## Abstract

**Background:** Cardiac arrest is a life-threatening event that requires early recognition and timely intervention. In-hospital cardiac arrest (IHCA) may be preceded by clinical deterioration, making competent monitoring, escalation, and resuscitation response essential. Cardiopulmonary resuscitation (CPR) is a key life-saving intervention, but CPR knowledge and skills may decline when healthcare professionals have limited opportunities for practice or exposure to real CPR events. **Aim:** This study aimed to evaluate CPR competency retention and compare knowledge and skills outcomes between nurses working in critical care and general care units. **Methods and Results:** A prospective comparative observational cohort study was conducted among 265 registered nurses. All participants were assessed immediately after BLS training, and each participant was reassessed once at an assigned post-training interval of 1, 3, or 6 months. Therefore, the findings represent subgroup comparisons across reassessment intervals rather than individual longitudinal trajectories across all time points. Knowledge was analyzed as a percentage score, while practical skills were analyzed categorically as Pass or Needs Remediation. Lower knowledge scores were observed at the assigned post-training reassessment intervals than at the immediate post-training assessment in both clinical groups, with clearer differences across reassessment intervals in the general care group. Skills competency also showed a higher proportion of nurses needing remediation at later intervals, particularly in the general care group. **Conclusions:** The findings suggest that CPR competency retention may vary across clinical settings and post-training reassessment intervals. The results support targeted refresher strategies, while acknowledging that causal explanations and individual longitudinal trajectories cannot be inferred from this design.

## 1. Introduction and Background

Cardiac arrest is defined as a sudden and unexpected stop of cardiac activity, resulting in a decrease in perfusion of blood to the vital organs, leading to an unresponsive victim with abnormal breathing and absence of signs of circulation [1]. In-hospital cardiac arrest (IHCA) is the most urgent medical emergency and may lead to death when not recognized and treated with prompt initiation and appropriate interventions by competent providers [2]. Generally, hospitals in the United States have been estimated to treat approximately 200,000 cardiac arrests annually [3].

Unlike some out-of-hospital cardiac arrests, IHCA is frequently preceded by clinical deterioration, including respiratory, circulatory, or neurological changes. Therefore, early detection, timely escalation, and competent CPR response are essential components of patient safety and resuscitation systems.

Cardiopulmonary resuscitation (CPR) is an important life-saving medical intervention employed in response to cardiac arrest. It is designed to restore vital functions and maintain adequate blood flow to the essential organs when the heart suddenly stops beating effectively by chest compressions, ventilation and defibrillation [4]. In general, research has demonstrated that when appropriately competent healthcare professionals (HCPs) administer CPR, it can decrease the incidence of unsuccessful resuscitation [2,5]. This distinction is particularly relevant in oncology care settings, where cardiac arrest is associated with markedly lower survival rates and often prompts reconsideration of code status among patients with cancer [6].

The American Heart Association (AHA) is a leading authority in cardiac resuscitation, conducting research, providing guidelines, and preparing healthcare professionals (HCPs) to respond effectively in CPR situations [4]. While the 2020 AHA guidelines recommended recertification every two years, more recent 2025 resuscitation science and education updates continue to emphasize high-quality CPR, deliberate practice, feedback, and systems-based strategies to sustain resuscitation performance [7,8,9,10]. These updates reinforce that certification alone may not be sufficient to maintain competency in practice, particularly when exposure to CPR events is infrequent.

Research indicates that infrequent CPR events can erode HCPs’ confidence and competence, potentially leading to suboptimal outcomes for patients in need of CPR [5]. Some studies suggest the need for more frequent CPR skills training than the biennial requirement due to the risk of skills decay from lack of practice [11,12,13,14,15]. Cardiopulmonary resuscitation skills decay over time [16,17]. This is also supported by multiple studies demonstrating that CPR skills decline too soon after training [18,19,20]. Other studies have revealed that compression rate and depth skills may be retained at two months, three months, and six months [21,22]. The recent literature indicates that CPR skills can deteriorate within weeks, while knowledge retention may decline between one and six months after training.

Competency retention refers to the extent to which knowledge, technical skills, and performance behaviors acquired during training are sustained over time and remain available for clinical application. In CPR education, retention is clinically important because resuscitation performance is time-sensitive and may be affected by the interval since training, frequency of deliberate practice, quality of feedback, and exposure to real clinical events. Nurses in critical care units may have more frequent exposure to cardiac arrest risk, acute deterioration, monitoring systems, and resuscitation teamwork than nurses in general care units. However, empirical evidence comparing retention patterns across clinical areas remains limited.

The AHA has introduced the Resuscitation Quality Improvement program to help HCPs maintain CPR proficiency. This initiative involves frequent, low-volume training sessions, requiring participants to demonstrate CPR skills on a manikin every 90 days to prevent skill decay [23]. However, the optimal training intervals for different providers and clinical specialties remain uncertain. The frequency of CPR cases varies based on working units, with critical care units encountering them more often due to patient characteristics. Limited research exists on CPR competency retention among nurses in critical care and general care units.

Therefore, the knowledge gap addressed by this study is the limited evidence regarding whether CPR knowledge and practical skills retention differ between nurses working in critical care units and those working in general care units when assessed at different post-training intervals.

## 2. Method

### 2.1. Study Design

This study used a prospective comparative observational cohort design. All participants completed an immediate post-training assessment (Ti) after mandatory institutional BLS training and were subsequently reassessed once at one assigned follow-up interval (T1, T2, or T3). Therefore, the study compared independent subgroups across reassessment intervals rather than repeatedly following the same participants over time.

Participants were categorized into critical care and general care groups based on their work setting. The absence of a control group was justified because BLS training is a mandatory institutional patient-safety requirement, and withholding training from eligible nurses was not ethically or operationally appropriate. The design was therefore selected to evaluate retention patterns in a real clinical education context while minimizing repeated testing effects.

### 2.2. Study Objectives, Research Questions, and Outcomes

The primary objective of this study was to evaluate CPR knowledge retention across post-training reassessment intervals and to compare knowledge outcomes between nurses in critical care and general care units. The secondary objective was to assess practical CPR skills competency across reassessment intervals and between clinical groups.

The study was guided by the following research questions: (1) What are the CPR knowledge scores of nurses in critical care and general care units at immediate post-training assessment and at assigned reassessment intervals? (2) Are there differences in knowledge scores across reassessment intervals within each clinical group? (3) Are there differences in the proportion of nurses who pass or need remediation in practical CPR skills across reassessment intervals? The primary outcome was CPR knowledge score. The secondary outcome was practical CPR skills competency, reported as Pass or Needs Remediation.

### 2.3. Recruitment and Participants

Out of 300 RNs invited to participate in the study during the period from April 2022 to April 2023, 275 RNs agreed to participate. Ultimately, 265 RNs completed the study, with 10 participants being withdrawn. Eligibility criteria required RNs to work in either the critical care or general care unit and to have no physical or medical restrictions that would prevent safe participation in CPR skills testing. Participants were excluded if they were not working in the target clinical areas, were unable to complete the assigned reassessment, withdrew consent, or had restrictions preventing performance of chest compressions.

All participants received information about the research purpose, design, confidentiality, and voluntary nature, with the option to withdraw without consequences. Withdrawn participants were not included in the final analysis because complete immediate and assigned follow-up assessment data were unavailable.

The target sample was estimated using an online sample size calculator based on the available nursing population who completed BLS training. With an estimated population of approximately 785 nurses, a 95% confidence level, 5% margin of error, and 50% response distribution, the minimum required sample was approximately 259 participants. The final analyzed sample of 265 nurses met this requirement.

### 2.4. Allocation and Data Collection

Prior to the BLS training course, participants were invited and consent was obtained. All participants received BLS training, and their initial assessment (Ti) was conducted immediately after the course. Participants were then allocated to one of the three post-training reassessment intervals: T1, T2, or T3. Allocation was performed after enrollment to distribute participants across the planned reassessment time points. Because this was an education-based clinical study conducted within routine workforce scheduling, allocation concealment was limited; however, scheduling was coordinated with unit managers to reduce participant preparation and minimize avoidable bias.

Certified AHA BLS instructors conducted all courses, with permission from the AHA to use the knowledge examination and skills evaluation checklist following the applicable AHA guidelines. Measures were taken to secure the exam according to training center policy.

The AHA BLS written examination consists of 25 multiple-choice questions with a total possible score of 100. To pass the exam, a mark of 84 must be achieved, allowing a maximum of four mistakes. The same examination was used for all participants at the immediate and post-training evaluations.

Practical CPR skills were assessed separately using the standardized AHA BLS skills testing checklist and the same scenario for all participants. Each required criterion was evaluated individually, and failure to meet any required criterion resulted in a final skills decision of Needs Remediation (NR). Therefore, the skills component was not converted into a percentage score or combined with the written examination score; knowledge and skills were analyzed as separate outcomes.

The 2020 AHA skills competency checklist contains three major areas of evaluation: initial assessment, high-quality CPR performance, and automated external defibrillator (AED) use. The high-quality CPR component included full chest recoil, minimized interruptions, compression depth of at least 5 cm, and compression rate of 100–120 compressions per minute. High-fidelity CPR manikins were used to support objective feedback on chest compression rate, depth, recoil, and interruptions and to reduce subjectivity in skills evaluation.

Skills assessments were conducted by certified AHA BLS instructors familiar with the standardized checklist and scenario. Evaluators used the same scenario, completion criteria, and manikin feedback indicators across assessment sessions. Formal inter-rater reliability testing was not conducted, which is acknowledged as a study limitation.

### 2.5. Ethical Considerations

The Institutional Review Board at King Hussein Cancer Center granted ethical approval for this study in accordance with the Declaration of Helsinki guidelines, approval number (22 KHCC 15). Participants were fully informed about the study purpose, methodology, and data management. They were assured of voluntary participation and the right to withdraw at any time. Informed consent was obtained from each participant prior to enrollment. Each participant received a unique participation code, and strict confidentiality and privacy measures were upheld throughout the study.

### 2.6. Data Analysis

Data analysis was conducted using SPSS version 29. Quantitative variables were summarized using means and standard deviations, and categorical variables were summarized using frequencies and percentages. Knowledge scores were analyzed as continuous variables, while skills competency was analyzed as a categorical outcome: Pass versus Needs Remediation. Independent subgroup comparisons across T1, T2, and T3 were conducted using one-way analysis of variance (ANOVA) for knowledge scores, with Bonferroni post hoc comparisons where appropriate. Chi-square tests were used to compare categorical skills outcomes across time-point subgroups. Because participants were reassessed at only one assigned follow-up interval, paired *t*-tests were not used for comparisons across T1, T2, and T3.

Assumptions for parametric testing were considered, including independence of observations, approximate normality of knowledge scores, and homogeneity of variance. Statistical significance was established at *p* < 0.05. The results are interpreted as subgroup comparisons across reassessment intervals rather than individual longitudinal change across all time points.

## 3. Results

### 3.1. Sociodemographic Characteristics

Table 1 presents the sociodemographic characteristics of the sample. The final sample included 265 registered nurses: 121 in critical care units and 144 in general care units. Gender and education distributions were not statistically different across time-point subgroups within the two clinical groups. Most participants held a bachelor’s degree, and most were female. Critical care nurses had a lower mean age and fewer mean years of professional experience compared with general care nurses. These baseline differences were considered when interpreting between-group findings.

### 3.2. CPR Knowledge Evaluation

**Figure 1:**
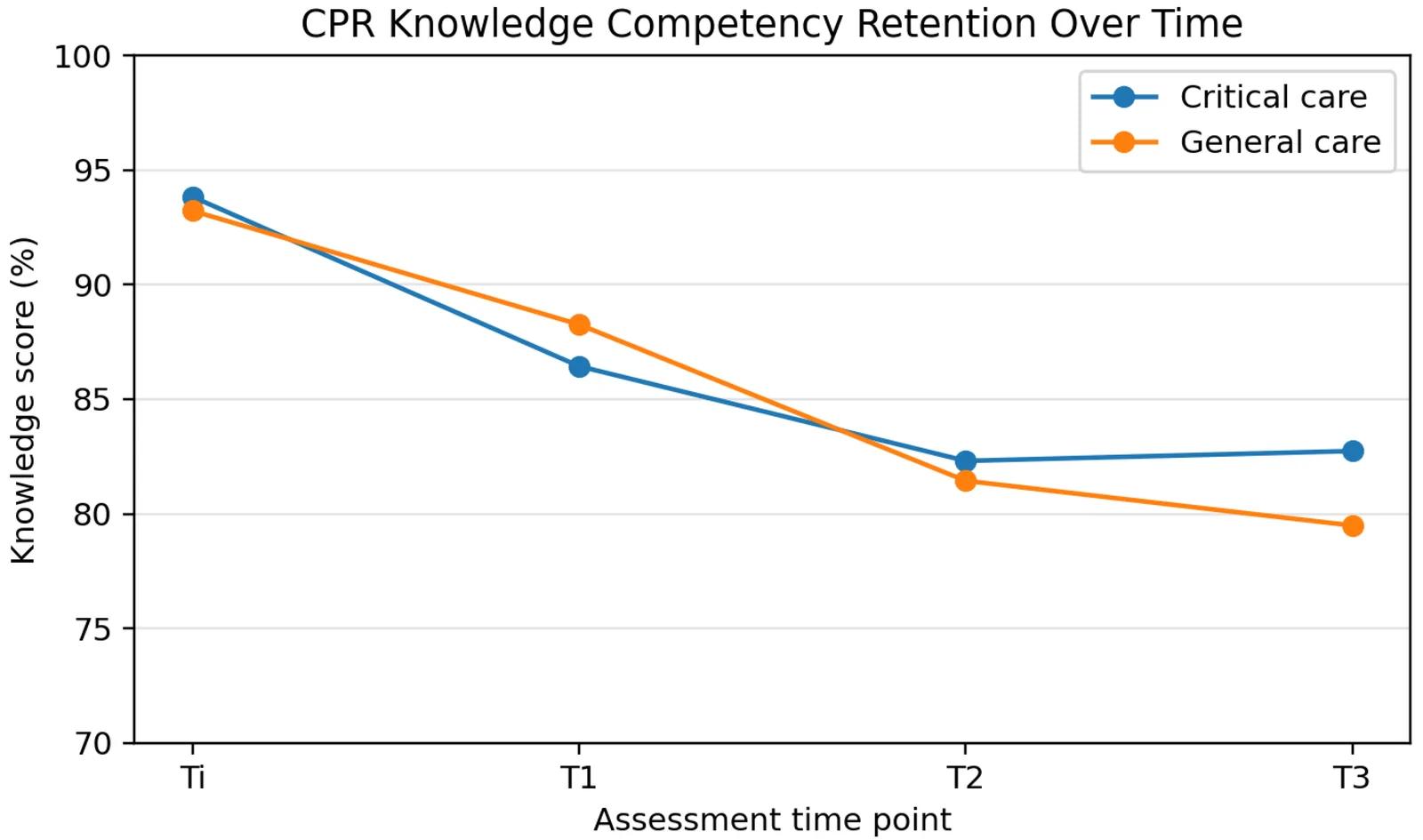
CPR Knowledge Competency Scores Across Assessment Points.

Knowledge scores were analyzed separately from practical skills decisions. Figure 1 and Table 2 summarize immediate post-training knowledge scores (Ti) and assigned post-training reassessment scores at T1, T2, and T3. In both clinical groups, mean knowledge scores were lower at reassessment compared with the immediate post-training assessment. However, because each participant was reassessed once at only one assigned interval, these results should be interpreted as subgroup comparisons rather than individual repeated trajectories.

The critical care group showed no statistically significant differences in reassessment knowledge scores across T1, T2, and T3 (*p* = 0.121). In contrast, the general care group showed statistically significant differences in reassessment knowledge scores across time-point subgroups (*p* < 0.001). As shown in Table 3, Bonferroni comparisons indicated that general care nurses assessed at T1 had significantly higher knowledge scores than those assessed at T2 and T3, while the T2 and T3 subgroups did not differ significantly.

### 3.3. CPR Skills Competency Evaluation

**Figure 2:**
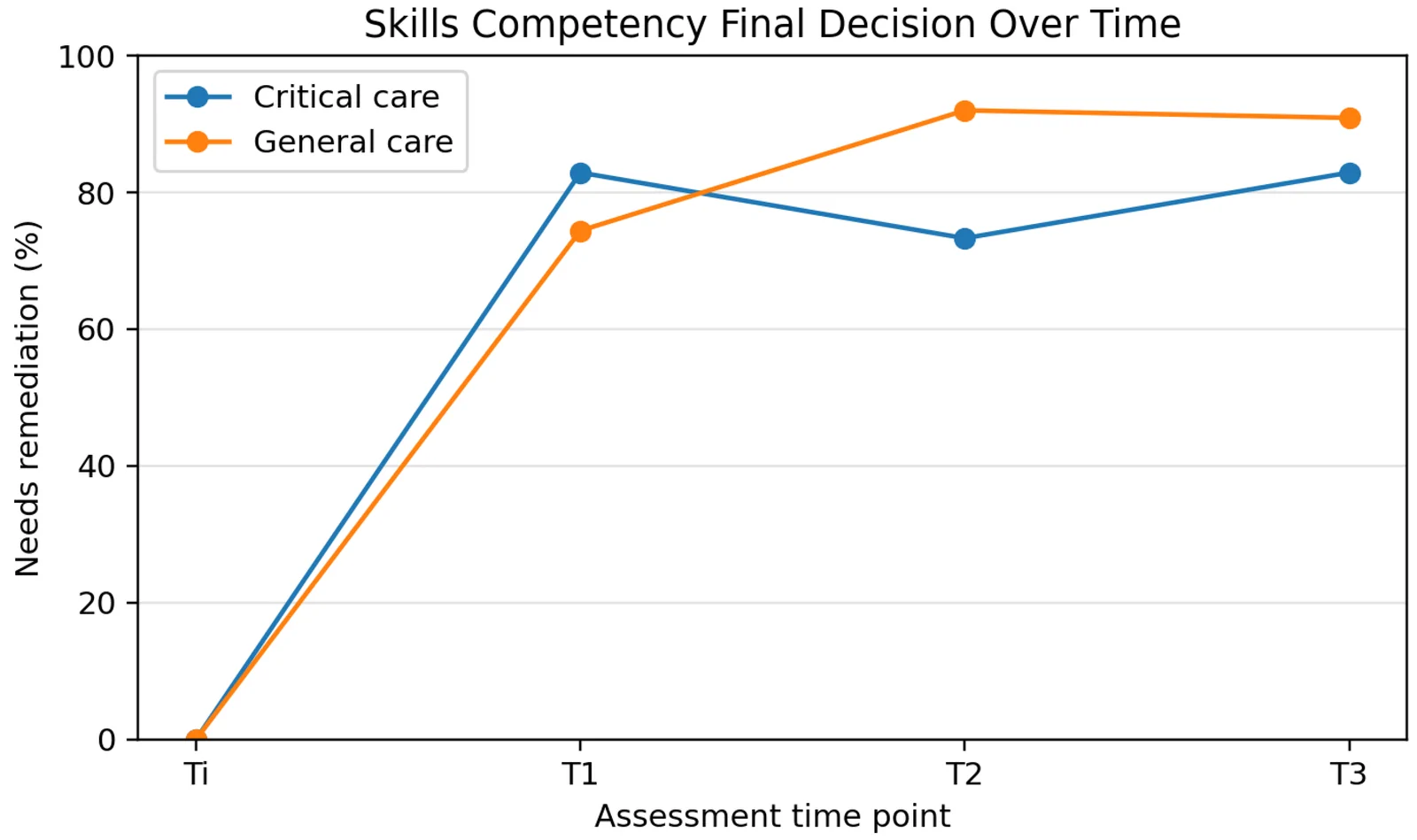
Percentage of Participants Needing Remediation in Practical CPR Skills at Reassessment.

Practical CPR skills were evaluated as a categorical final decision: Pass or Needs Remediation (NR). At the immediate post-training assessment (Ti), all participants passed the skills evaluation. Table 4 and Figure 2 present post-training reassessment decisions across time-point subgroups. In the critical care group, the proportion requiring remediation was 82.9% at T1, 73.3% at T2, and 82.9% at T3. In the general care group, the proportion requiring remediation was 74.4% at T1, 92.0% at T2, and 90.9% at T3.

No statistically significant differences were observed across the critical care reassessment subgroups. In the general care group, significant differences were observed across reassessment intervals, with higher remediation proportions at T2 and T3 compared with T1. These findings suggest that general care nurses may require earlier or more frequent refresher support; however, causal explanations cannot be confirmed without individual longitudinal follow-up and data on actual CPR exposure during the follow-up period.

## 4. Discussion

The purpose of this study was to evaluate CPR competency retention among registered nurses and to compare knowledge and practical skills outcomes between critical care and general care units. The findings showed lower knowledge scores at post-training reassessment compared with immediate post-training assessment and a high proportion of participants requiring remediation in practical skills at later reassessment intervals. These findings are consistent with the previous literature indicating that CPR knowledge and skills may decline over time without deliberate practice, feedback, and reinforcement [15,24].

### 4.1. CPR Knowledge and Skills After Initial Training

Immediately after training, participants achieved passing performance in practical skills, supporting the effectiveness of standardized BLS training and instructor-led assessment. However, differences observed at later reassessment intervals should be interpreted cautiously because this study compared subgroups assessed at different time points rather than tracking the same participants across all intervals. The lower reassessment knowledge scores and high remediation rates highlight the challenge of sustaining CPR competency after initial certification.

Critical care nurses had a slightly higher immediate knowledge score and generally showed less pronounced differences across reassessment intervals than general care nurses. A plausible explanation is that critical care nurses may have greater exposure to high-acuity patient deterioration, monitoring systems, emergency response procedures, and resuscitation team activities. However, actual exposure to CPR events during follow-up was not measured; therefore, this explanation should be considered interpretive rather than confirmed by the data.

The study found that RNs in the critical group were generally younger than those in the general group. Previous studies have suggested that age, the nature of critical care work, and CPR frequency may influence information acquisition and retention [25,26,27]. However, because the present study did not evaluate age as an independent predictor and did not perform adjusted analyses, age and professional experience should be considered baseline characteristics and potential confounders rather than explanatory factors.

Regarding skills competency, all participants in both groups passed the skills evaluation immediately after CPR training, in line with previous research emphasizing the effectiveness of CPR training using high-fidelity manikins and standardized methods [24,28,29].

### 4.2. CPR Knowledge and Skills Decay at Different Time Intervals

The present findings indicate that both groups experienced knowledge and skills decay after initial CPR training, consistent with the existing literature [3,15,24,29,30]. Many participants did not maintain optimal CPR competencies over time, leading to the need for remediation. This was also reported in similar studies [31].

The general care group showed statistically significant differences in knowledge scores across reassessment intervals, with higher scores at T1 than at T2 and T3. In contrast, the critical care group did not show statistically significant differences in reassessment knowledge scores across T1, T2, and T3. For practical skills, the general care group also showed significant differences in remediation proportions across intervals, whereas the critical care group did not. These findings suggest that competency retention may differ by clinical area, but the study design does not allow definitive claims about individual deterioration trajectories.

The results have practical implications for CPR education. Rather than relying only on biennial recertification, clinical educators may consider targeted low-dose, high-frequency refresher activities, manikin-based feedback, and unit-specific competency maintenance strategies. Training programs may also consider risk-informed refresher strategies tailored to clinical area, baseline competency, likelihood of CPR exposure, and previous remediation needs. General care nurses may particularly benefit from scheduled refresher sessions because they may have fewer opportunities to participate in real resuscitation events. Critical care nurses may also require structured refreshers, but the frequency and emphasis may differ according to unit exposure, risk profile, and institutional resuscitation priorities.

These findings align with contemporary resuscitation education recommendations that emphasize deliberate practice, feedback, and systems-based improvement. The 2025 AHA and ILCOR updates further support the need to move beyond certification as a single event and to consider strategies that sustain readiness and performance over time [7,8,9,10].

### 4.3. Strengths and Limitations

This study has several strengths. It addresses a clinically important topic in nursing education and patient safety, includes a relatively large sample of registered nurses from both critical care and general care units, and uses standardized AHA knowledge and skills assessment tools. The use of high-fidelity CPR manikins strengthened the objectivity of practical skills assessment, particularly for compression rate, depth, recoil, and interruptions.

Several limitations should also be acknowledged. First, this was a single-center study, which may limit generalizability to other institutions or healthcare systems. Second, participants were reassessed at only one assigned post-training interval; therefore, the findings represent subgroup comparisons rather than individual longitudinal trajectories. Third, actual exposure to CPR events during the follow-up period was not measured, limiting the ability to directly link clinical exposure with retention. Fourth, baseline differences in age and professional experience between clinical groups may have influenced the findings, and multivariable adjusted analyses were not performed. Fifth, formal inter-rater reliability testing among skills assessors was not conducted. Finally, lower scores on a theoretical examination may not necessarily represent poorer performance in actual CPR situations, where teamwork, situational awareness, leadership, and environmental factors also influence performance.

## 5. Conclusions

This study suggests that CPR knowledge and practical skills competency differ across post-training reassessment intervals among registered nurses and that retention patterns may vary between critical care and general care units. General care nurses demonstrated clearer differences across reassessment intervals, particularly in knowledge scores and the proportion requiring skills remediation. However, these findings should be interpreted as subgroup comparisons rather than individual longitudinal deterioration. Differences between clinical groups may be related to clinical exposure and organizational context, but these factors were not directly measured. The findings support the need for tailored refresher training, ongoing competency monitoring, and future multi-center studies using longitudinal follow-up, measured CPR exposure, and adjusted statistical models.

## Figures and Tables

**Table 1:** Sociodemographic Characteristics of the Sample.

Variable	Critical T1 (*n* = 41)	Critical T2 (*n* = 45)	Critical T3 (*n* = 35)	General T1 (*n* = 39)	General T2 (*n* = 50)	General T3 (*n* = 55)
Male, n (%)	16 (39.0)	16 (35.6)	10 (28.6)	11 (28.2)	12 (24.0)	17 (30.9)
Female, n (%)	25 (61.0)	29 (64.4)	25 (71.4)	28 (71.8)	38 (76.0)	38 (69.1)
BSc, n (%)	35 (85.4)	42 (93.3)	35 (100)	38 (97.4)	48 (96.0)	50 (90.9)
Master, n (%)	6 (14.6)	3 (6.7)	0 (0.0)	1 (2.6)	2 (4.0)	5 (9.1)
Age, mean (SD)	26.62 (5.84)			28.81 (6.71)		
Experience, mean (SD)	4.53 (4.98)			6.47 (5.92)		

Note. Gender and education comparisons were not statistically significant within clinical groups. Age and working experience differed significantly between critical care and general care nurses and were considered in interpreting the findings.

**Table 2:** Knowledge Competency Scores by Clinical Group and Reassessment Interval.

Group	Assessment	T1 n	T1 Mean (SD)	T2 n	T2 Mean (SD)	T3 n/Mean (SD)
Critical	Immediate Ti	41	95.22 (4.92)	45	92.40 (4.29)	35/94.06 (3.55)
Critical	Reassessment	41	86.44 (10.65)	45	82.31 (10.22)	35/82.74 (8.56)
General	Immediate Ti	39	92.41 (4.18)	50	93.12 (5.83)	55/93.89 (4.98)
General	Reassessment	39	88.26 (7.46)	50	81.44 (11.10)	55/79.49 (12.95)

Note. Knowledge scores are reported as percentages out of 100. T1 = 1 month; T2 = 3 months; T3 = 6 months. The table presents subgroup reassessment comparisons, not repeated follow-up of all participants across all time points.

**Table 3:** Non-Redundant Bonferroni Pairwise Comparisons of Reassessment Knowledge Scores.

Group	Comparison	Mean Difference	Std. Error	*p* Value	95% CI Lower	95% CI Upper
Critical	T1 vs. T2	4.13	2.14	0.169	−1.08	9.33
Critical	T1 vs. T3	3.70	2.28	0.325	−1.85	9.24
Critical	T2 vs. T3	−0.43	2.24	1.000	−5.86	5.00
General	T1 vs. T2	6.82	2.36	0.013	1.10	12.54
General	T1 vs. T3	8.77	2.31	<0.001	3.16	14.37
General	T2 vs. T3	1.95	2.16	1.000	−3.28	7.18

Note. Only non-redundant pairwise comparisons are presented to improve readability. Positive mean differences indicate higher scores in the first listed subgroup.

**Table 4:** Practical Skills Competency Final Decision by Group and Reassessment Interval.

Group	Time Point	Total n	Passed, n (%)	Needs Remediation, n (%)	Statistical Comparison
Critical	T1	41	7 (17.1)	34 (82.9)	Across T1–T3: *p* = 0.455
Critical	T2	45	12 (26.7)	33 (73.3)	T1 vs. T2: *p* = 0.311
Critical	T3	35	6 (17.1)	29 (82.9)	T1 vs. T3: *p* = 0.994
General	T1	39	10 (25.6)	29 (74.4)	Across T1–T3: *p* = 0.026
General	T2	50	4 (8.0)	46 (92.0)	T1 vs. T2: *p* = 0.038
General	T3	55	5 (9.1)	50 (90.9)	T1 vs. T3: *p* = 0.045

Note. Ti is not displayed because all participants passed immediately after training. NR = Needs Remediation. Skills competency was not converted into a percentage score and was not combined with the knowledge score.

## Data Availability

The original data presented in the study are openly available in Figshare at https://figshare.com/s/9d157697e1f4b4794ca5 (accessed on 16 July 2026).

## Public Involvement Statement

No public involvement in any aspect of this research.

## Guidelines and Standards Statement

This manuscript was drafted in accordance with the STROBE (Strengthening the Reporting of Observational Studies in Epidemiology) Statement for observational studies.
